# Simultaneous Bilateral Optic Neuritis Following Human Papillomavirus Vaccination in a Young Child

**DOI:** 10.7759/cureus.3352

**Published:** 2018-09-24

**Authors:** Ngu Dau Bing Michael, Tengku Norina Tuan Jaffar, Adil Hussein, Wan-Hazabbah Wan Hitam

**Affiliations:** 1 Ophthalmology, School of Medical Sciences/Universiti Sains Malaysia, Kelantan, MYS; 2 Ophthalmology, Raja Perempuan Zainab Il Hospital, Kelantan, MYS; 3 Ophthalmology, School of Medical Sciences/Universiti Sains Malaysia, Kota Bharu, MYS

**Keywords:** optic neuritis, human papillomavirus, vaccination

## Abstract

Vaccination-induced optic neuritis is not common. The development of optic neuritis following various vaccinations have been reported, suggesting a possible association between optic neuritis and vaccination. Of those reported cases, influenza vaccines have been the most common. Although rare, those patients who developed optic neuritis following HPV vaccination also presented with other central nervous system (CNS) demyelinating syndromes, especially following a booster dose. We present a rare case of simultaneous isolated bilateral optic neuritis following the first dose of an HPV vaccination in a young child. She received treatment with a systemic corticosteroid that resulted in a good clinical outcome without developing any demyelinating disease.

## Introduction

Optic neuritis is an inflammation of the optic nerve which can be caused by demyelination, infection, post-immunization, or autoimmune diseases. The most common cause of optic neuritis in children is a viral infection. Hence, it seems possible that vaccines can also cause a similar condition by stimulating the host's immune system. The exact mechanism is unknown, but it is believed that the activation of T-cells may cross the blood-brain barrier, resulting in a type IV hypersensitivity reaction that damages the myelin sheath of optic nerve [1]. Presentation of optic neuritis in children has several unique characteristics, such as it is more common to have optic disc swelling and more often bilateral simultaneous condition. They usually have a good prognosis and less association with multiple sclerosis [2]. A few case reports have suggested a possible association between the demyelinating syndrome and the human papillomavirus (HPV) vaccination [3-8]. For patients with HPV vaccination-induced optic neuritis, they also developed other central nervous system (CNS) demyelinating syndromes during the presentation or later in life. We present a rare case of simultaneously isolated bilateral optic neuritis following the first dose of HPV vaccination.

## Case presentation

**Figure 1:**
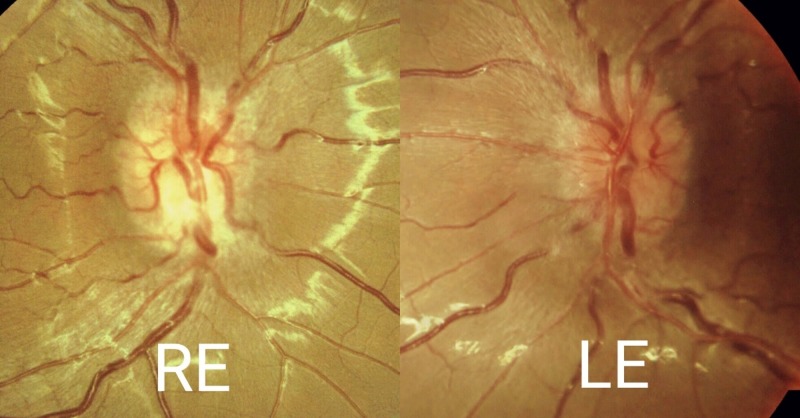
Funduscopy of the right and left eyes Right eye (RE) fundus photo showed the superior optic disc to be hyperemic and inferiorly slightly pale. Left eye (LE) optic disc was generally hyperemic.

**Figure 2:**
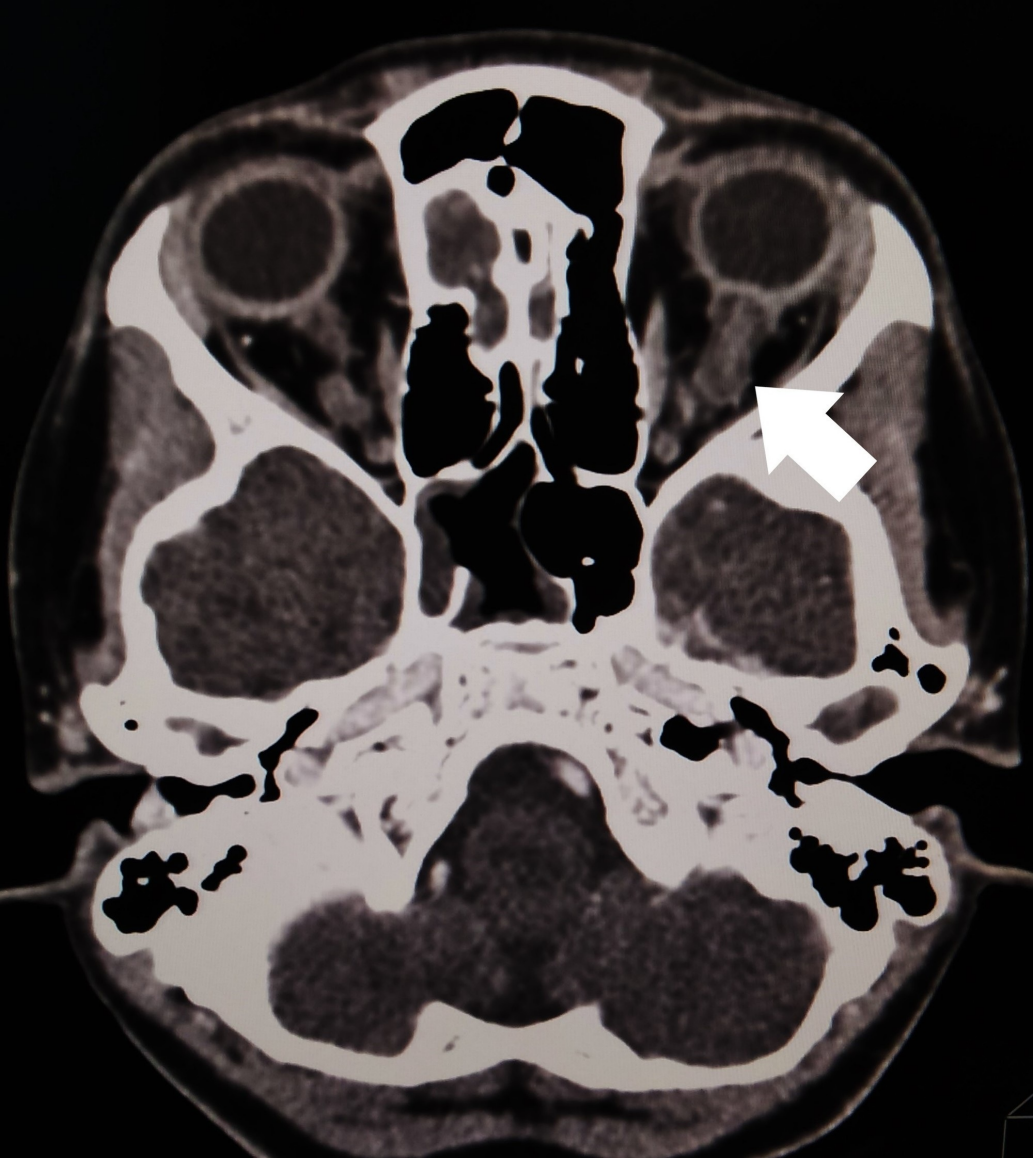
Computed tomography (CT) of the brain, orbit, and paranasal area Left optic nerve (arrow) is slightly prominent compared to the right side

**Figure 3:**
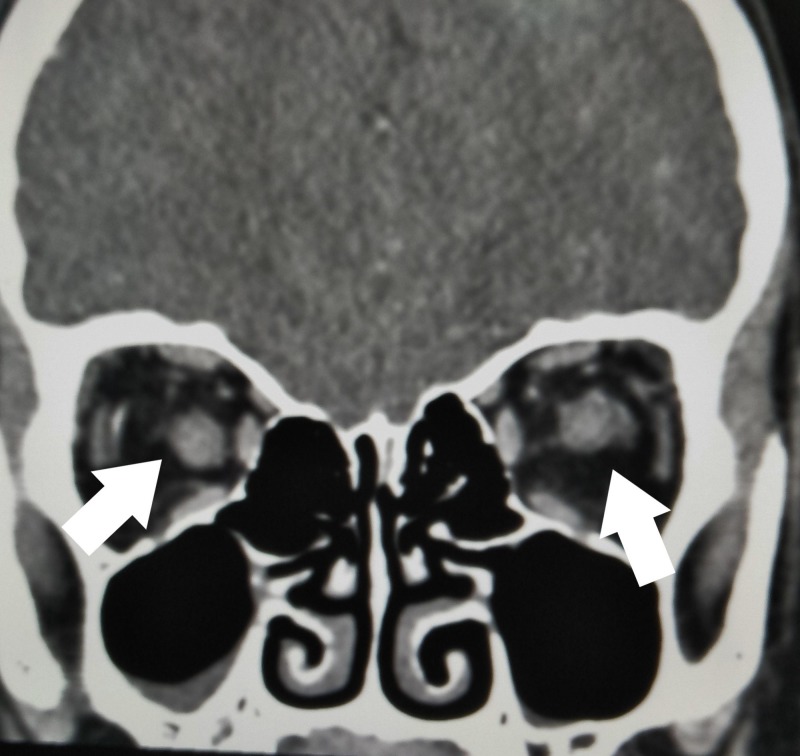
Computed tomography (CT) of the optic nerves Coronal view showing the left optic nerve is larger than right optic nerve (arrows)

A 13-year-old healthy female student with no medical illness presented with sudden onset loss of vision in both eyes for three days. It was associated with pain on eye movements. She had her first dose of an HPV vaccination three weeks prior to the presentation and reported having flu-like symptoms after the vaccination. There was no history of limb numbness, weakness, or walking difficulty. The bowel opening and micturition were normal. On examination, visual acuity in the right eye was counting fingers and left eye was hand movement. Both pupils were sluggish and a relative afferent pupillary defect (RAPD) was not apparent. Both anterior segments were unremarkable. Funduscopy showed a diffuse hyperemic swollen disc with dilated vessels in both eyes. The left eye was found to be more profound than the right eye (Figure 1). The macula was normal in both eyes. There was no sign of retinitis or choroiditis. She was admitted to the ward for further investigation. Urgent computed tomography (CT) of the brain, orbit, and paranasal area was performed. Both optic nerves had homogenous enlargement which was suggestive of optic neuritis. The left optic nerve was slightly prominent compared to the right side (Figures 2-3). The brain and paranasal views were normal. An erythrocyte sedimentation rate (ESR) showed 6 mm/hour and the white cell count was within normal limit. Blood screening for infective and autoimmune processes was unremarkable. Mantoux test was negative. A diagnosis of bilateral optic neuritis post-HPV vaccination was made. She was treated with intravenous methylprednisolone, 125 mg four times a day (qid) for three days, followed by oral prednisolone 1 mg/kg/day for 11 days. Her visual acuity improved significantly to 6/6 bilaterally after one week of treatment. Fundus examination showed healthy optic discs. Optic nerve functions were normal. She has been under eye clinic follow-up for two years and does not show any sign of CNS demyelinating syndrome.

## Discussion

Numerous case reports have suggested an association between CNS demyelinating syndrome and several different vaccines [3-4]. Of that, influenza vaccines were the most commonly reported, probably due to the H1N1 outbreak between 2009 and 2012 [3-4]. Few cases of such adverse drug reactions have also been reported for HPV vaccines [5-8]. Based on the literature review, the symptoms tend to occur after a booster dose rather than first inoculation [5, 7-8]. Moreover, apart from optic neuritis, they also presented with other clinical syndromes, such as neuromyelitis optica (NMO), myelitis, or acute disseminated encephalomyelitis (ADEM) [3-8]. However, for the patient in this case report, her case was relatively unusual as she developed simultaneous isolated bilateral optic neuritis after the first dose of the HPV vaccination. Although she has been under clinic follow-up for two years, there was no evidence of developing a neurodegenerative or demyelinating disease.

The mechanism of this pathology is still unknown but is presumably immune-mediated. Activation of T-cells secondary to an inflammatory process may cross the blood-brain barrier and induce a type IV hypersensitivity reaction resulting in the destruction of the myelin sheath of the nerves [1]. In vaccines, the presence of adjuvants that induce an immune response is believed to cause activation of the autoimmune syndrome [3-4]. However, it is hard to determine whether the occurrence of the optic neuritis was coincidence or caused by the vaccination, especially when it followed the first dose of HPV vaccination shortly thereafter. To our knowledge, there is only one other case report identifying optic neuritis after the first dose of HPV vaccination. That patient also developed recurrent optic neuritis after a booster dose and was finally diagnosed with NMO spectrum disease [6].

Pediatric optic neuritis is commonly bilateral and occurs after a viral infection or immunization. It is a clinical diagnosis, according to findings, that includes visual loss, pain on ocular movement, RAPD, dyschromatopsia, and the appearance of the optic disc [2]. However, one needs to exclude other causes, such as infection, a demyelinating lesion, or autoimmune disease, through laboratory and imaging investigations. As there is a possible association between optic neuritis and multiple sclerosis, magnetic resonance imaging (MRI) of the brain can help to detect a demyelinating lesion [2]. However, for this patient, a CT of the brain (rather than an MRI) was done due to hospital limitations, especially during the acute setting.

No clinical trials have been done for optic neuritis in children. Conservative management is the treatment of choice for unilateral or mild bilateral optic neuritis [2]. For bilateral cases with a severe visual loss, treatment is based on optic neuritis treatment trial [9]. By treating with intravenous corticosteroids, it helps to hasten the recovery of vision, although the final visual acuity is unaffected [10]. A better visual prognosis is seen in those with solitary optic neuritis than other demyelinating diseases [3-4]. This was also seen in this patient whose vision recovered to 6/6 bilateral after the treatment. However, she has needed to be on long-term follow-up for monitoring of demyelinating disease.

To date, there is no conclusive evidence of HPV vaccination in the pathogenesis of demyelinating diseases. For this patient, she refused the booster dose of the HPV vaccination. The decision for the continuation of the HPV vaccination is a personal decision and is not compulsory. Hence, all the patients who are scheduled for HPV vaccination should be informed of optic neuritis as one of the possible adverse events. However, reviews over the safety of HPV vaccines has shown that it is generally safe and well-tolerated [11-12].

## Conclusions

Isolated optic neuritis following the first dose of an HPV vaccination is very rare. Response to corticosteroids treatment is promising. However, infective causes need to be ruled out. Longer follow-up is required to detect early demyelinating disease.
